# Rapidly progressive Hodgkin lymphoma causing superior vena cava syndrome in late pregnancy: a case report and literature review

**DOI:** 10.3389/fmed.2026.1786362

**Published:** 2026-04-29

**Authors:** Branka Čančarević-Đajić, Zora Antonić, Stefan Božić, Marko Kantar, Amela Cerić, Dijana Mikić, Svetlana Tomašević-Pavlović, Predrag Berić

**Affiliations:** 1 Clinic of Gynecology and Obstetrics, University Clinical Center of the Republic of Srpska, Banja Luka, Bosnia and Herzegovina; 2 Clinic of Thoracic Surgery, University Clinical Center of the Republic of Srpska, Banja Luka, Bosnia and Herzegovina; 3 Clinic of Hematology, University Clinical Center of the Republic of Srpska, Banja Luka, Bosnia and Herzegovina; 4 Clinic of Radiology, University Clinical Center of the Republic of Srpska, Banja Luka, Bosnia and Herzegovina; 5 Department of Clinical Pathology, University Clinical Center of the Republic of Srpska, Banja Luka, Bosnia and Herzegovina; 6 Clinic of Anesthesiology and Intensive Care Medicine, University Clinical Center of the Republic of Srpska, Banja Luka, Bosnia and Herzegovina

**Keywords:** ABVD chemotherapy, Hodgkin lymphoma, interleukin-6, mediastinal mass, pregnancy, superior vena cava syndrome

## Abstract

Hodgkin lymphoma during pregnancy is rare and may present diagnostic and therapeutic challenges, particularly when complicated by superior vena cava syndrome, a life-threatening condition. We report a 41-year-old pregnant patient at 30 weeks of gestation who presented with rapidly progressive dyspnea, cervical venous congestion, and facial edema. Imaging revealed a bulky mediastinal mass compressing the superior vena cava. Due to acute maternal risk, an emergency multidisciplinary decision was made to perform cesarean delivery followed by immediate uniportal video-assisted thoracoscopic biopsy. Histopathology confirmed classical Hodgkin lymphoma, nodular sclerosis subtype. Postpartum positron emission tomography/computed tomography demonstrated extensive supradiaphragmatic disease, and chemotherapy with doxorubicin, bleomycin, vinblastine, and dacarbazine was promptly initiated. The patient showed a favorable clinical and metabolic response, and the neonate demonstrated normal early postnatal development. This case highlights the importance of early recognition and multidisciplinary management of superior vena cava syndrome in pregnancy and raises the hypothesis that pregnancy-associated immunologic changes, including interleukin-6–mediated pathways, may contribute to accelerated disease progression.

## Introduction

Hodgkin lymphoma (HL) is a malignant lymphoproliferative disorder accounting for approximately 10% of all lymphomas and 0.2% of cancer-related mortality worldwide (1). Although rare during pregnancy, with an estimated incidence of 1 in 1,000–6,000 pregnancies, HL represents the most commonly diagnosed lymphoma in pregnant women (2). Management is particularly challenging, as optimal maternal treatment must be balanced against fetal safety.

Superior vena cava syndrome (SVCS) is an uncommon but potentially fatal complication of mediastinal lymphomas. During pregnancy, physiological hypervolemia, reduced venous return, and cardiopulmonary adaptations may exacerbate venous obstruction and accelerate clinical deterioration (3–5). Diagnostic limitations and anesthetic considerations further complicate management.

We present a rare case of rapidly progressive classical Hodgkin lymphoma manifesting as SVCS in the third trimester of pregnancy, successfully managed through urgent cesarean delivery, simultaneous diagnostic thoracoscopic biopsy, and prompt initiation of systemic therapy.

## Case presentation

A 41-year-old woman (gravida 3, para 1) at 30 + 1 weeks of gestation presented with a 7-day history of progressive shortness of breath, neck swelling, facial fullness, generalized weakness, malaise, and reduced appetite. She denied chest pain, headache, visual disturbances, or vaginal bleeding. There was no family history of malignancy; however, her father had a history of chronic heart failure and renal insufficiency. The patient was a non-smoker and denied alcohol consumption or exposure to environmental risk factors. Her medical history included thyroid goiter and inherited thrombophilia, without anticoagulant therapy during pregnancy. The pregnancy had been regularly monitored and had progressed without complications.

On admission, the patient was conscious and oriented. Vital signs revealed tachycardia (111 bpm), blood pressure 110/60 mmHg, oxygen saturation 97% on room air, and eupnea at rest. Physical examination showed visible cervical venous distension and diminished breath sounds at the right lung base, without peripheral edema.

Laboratory findings demonstrated normocytic anemia (hemoglobin 102 g/L), elevated C-reactive protein (55.9 mg/L), and mildly elevated D-dimer (1.26 mg/L FEU), with preserved renal and hepatic function.

### Differential diagnosis and initial evaluation

Given the acute dyspnea and history of thrombophilia, pulmonary embolism, acute coronary syndrome, and myopericarditis were considered. Electrocardiography showed sinus tachycardia without ischemic changes. Transthoracic echocardiography revealed preserved ventricular function with a small circumferential pericardial effusion (up to 6 mm). Color Doppler ultrasonography of the neck vessels demonstrated dilated but patent jugular and subclavian veins without thrombosis, suggesting extrinsic compression rather than intravascular obstruction.

### Diagnostic imaging

**Figure 1:**
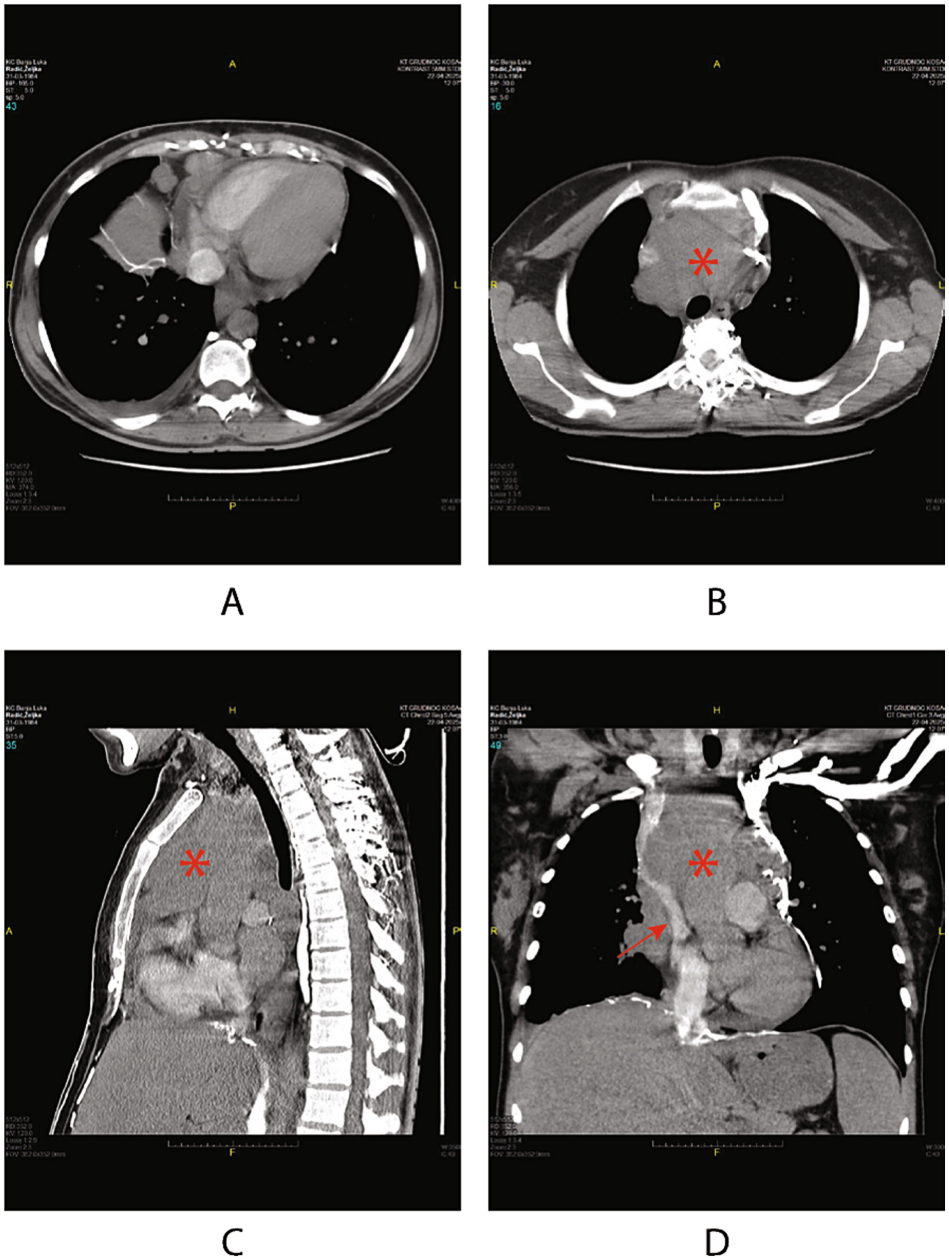
Contrast-enhanced chest CT images demonstrating superior vena cava syndrome. Panels **(A, B)** show axial sections, and panels **(C, D)** show sagittal reconstructions. CT of the thorax demonstrating a bulky mediastinal mass (*) causing extrinsic compression of the superior vena cava (arrow), consistent with superior vena cava syndrome.

Urgent contrast-enhanced chest computed tomography revealed bulky mediastinal lymphadenopathy forming conglomerates compressing the superior vena cava, with extensive collateral venous circulation, consistent with SVCS (Figures 1A–D). Additional findings included hepatosplenomegaly, cervical and retroperitoneal lymphadenopathy, and right pleural effusion.

The patient was transferred to the intensive care unit for close monitoring.

### Multidisciplinary management and surgery

Due to rapid clinical progression, significant venous obstruction, and fetal viability, a multidisciplinary team involving obstetrics, anesthesiology, thoracic surgery, hemato-oncologist, and neonatology recommended urgent delivery followed by immediate diagnostic intervention.

Cesarean section was performed under general anesthesia using the Misgav-Ladach technique, resulting in the delivery of a premature male neonate weighing 2,020 g, with Apgar scores of 9 and 9 at 1 and 5 min, respectively. The neonate was admitted to the neonatal intensive care unit for prematurity-related observation.

Immediately after delivery, uniportal video-assisted thoracoscopic surgery (VATS) biopsy of the mediastinal mass was performed. Multiple tissue samples were obtained, and a chest drain was placed.

### Histopathology

**Figure 2:**
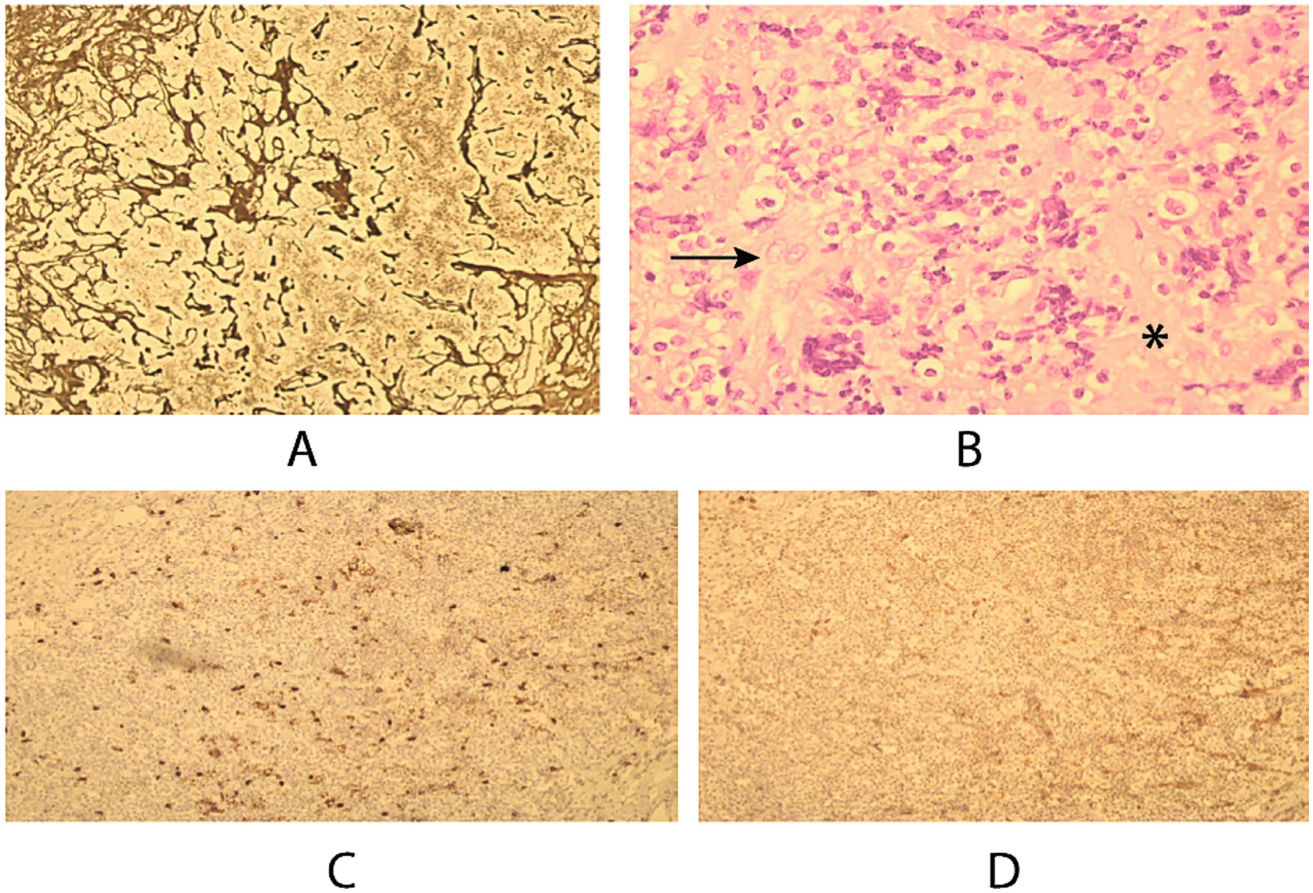
Histopathological characterization of the mediastinal lymph node. **(A)** Reticulin stain highlighting the fibrotic framework of the lymphoid tissue. **(B)** Hematoxylin and eosin staining showing lacunar-type Reed–Sternberg cells (arrow) and sclertoic bands (*). **(C)** Immunohistochemistry for CD15 demonstrating strong membranous positivity. **(D)** Immunohistochemistry for CD30 showing diffuse strong positivity, consistent with classical Hodgkin lymphoma.

Histopathological examination of the mediastinal lymph node biopsy demonstrated classical Hodgkin lymphoma, nodular sclerosis subtype, characterized by Reed–Sternberg and lacunar cells. Immunohistochemistry showed positivity for CD15, CD30, and MUM1, with weak or absent CD20 expression and negative Epstein–Barr virus status, confirming the diagnosis (Figure 2).

### Postoperative course, follow-up, and outcomes

**Figure 3:**
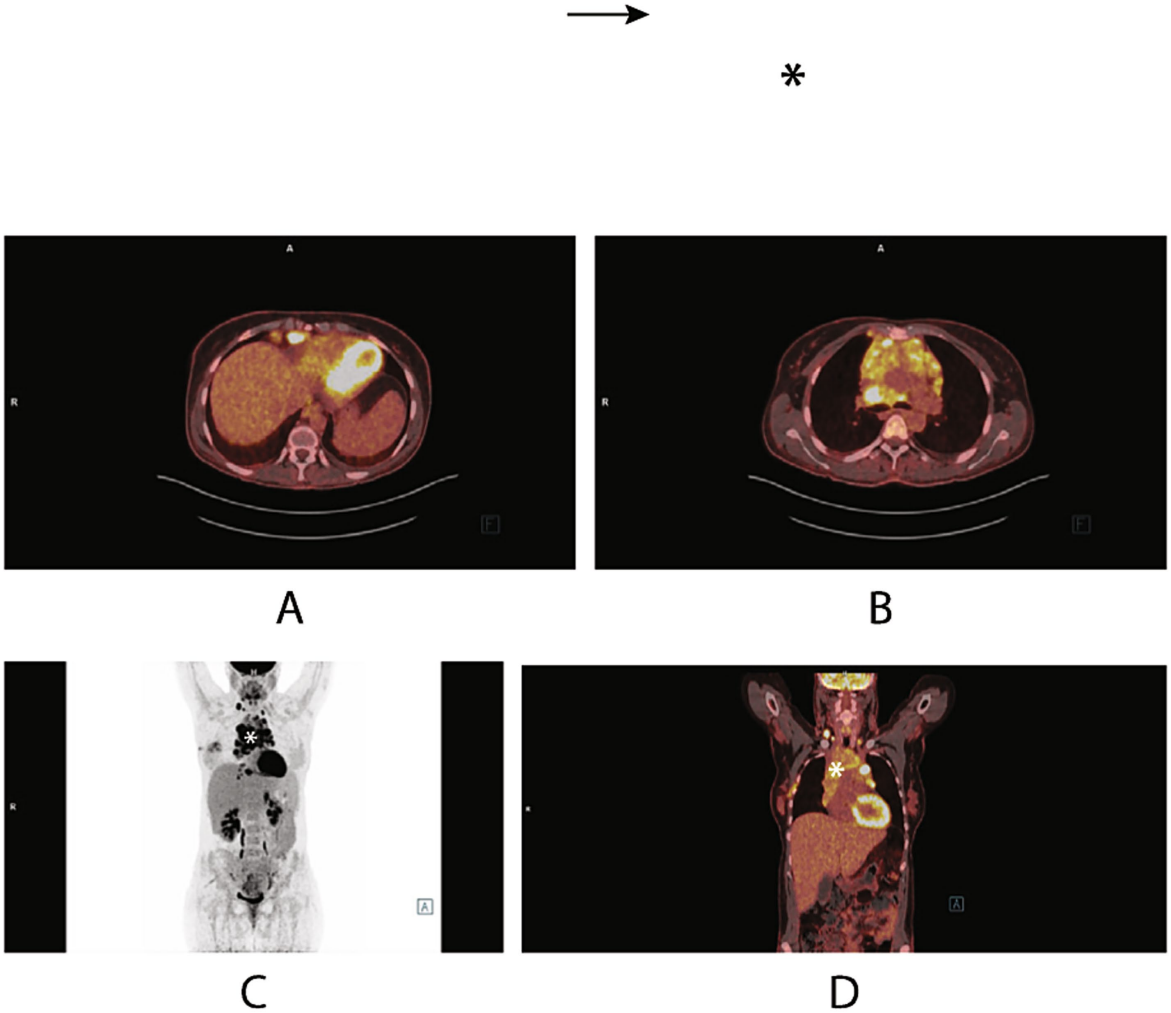
Postpartum PET/CT scan demonstrating metabolically active lymph nodes in the neck and a bulky mediastinal mass (*), consistent with lymphoproliferative disease. Additional focal uptake is seen in individual lymph nodes within the supradiaphragmatic paracardiac fat and preesophageal region. A nodule in the S3 segment of the right lung is noted but too small for characterization. Bilateral pleural effusions are present without FDG uptake. Hepatosplenomegaly is also observed.

**Figure 4:**
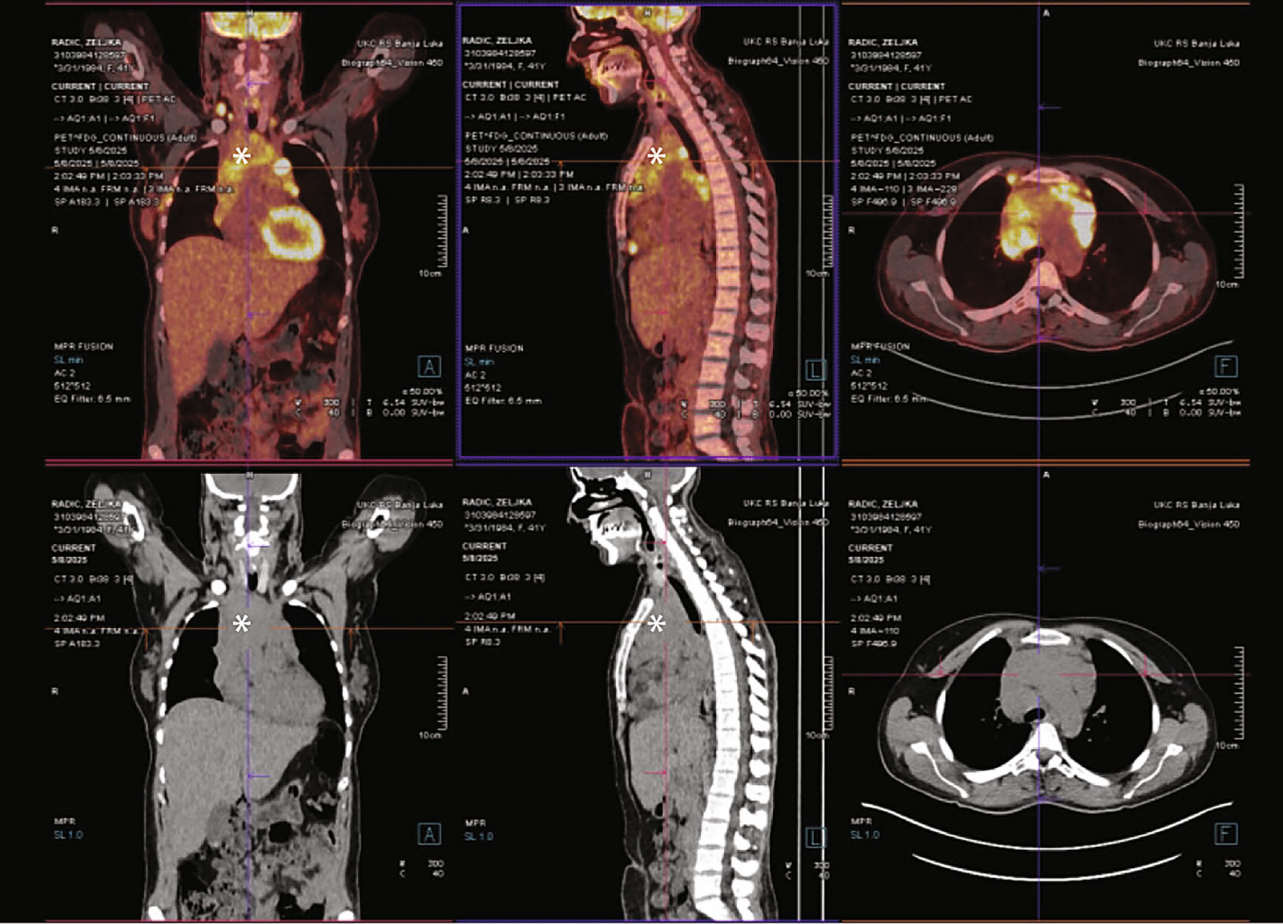
Comparison of contrast-enhanced CT and PET/CT images of the mediastinal mass (*). The CT images show bulky mediastinal lymphadenopathy, while the PET/CT images demonstrate intense FDG uptake in the same regions, highlighting metabolically active disease.

Fifteen days postpartum, the patient underwent an initial PET/CT scan to establish baseline disease burden. Imaging revealed metabolically active bulky mediastinal lymphadenopathy, with involvement of select supradiaphragmatic lymph nodes in the paracardial and paraesophageal regions, as well as a nodular lesion in the S3 segment of the right lung. Bilateral pleural effusions were present without metabolic activity, and mild hepatosplenomegaly was noted (Figures 3, 4).

Following this baseline assessment, the patient received two cycles of ABVD chemotherapy, after which a second PET/CT scan was performed to evaluate early response. Subsequent completion of four additional cycles (for a total of six cycles) was followed by post-treatment imaging. PET/CT demonstrated a marked reduction in mediastinal mass size, with residual metabolic activity in select cervical lymph nodes, prompting multidisciplinary recommendation for consolidative radiotherapy targeting the mediastinal bulk and cervical lymph nodes (36 Gy in 18 fractions).

**Figure 5:**
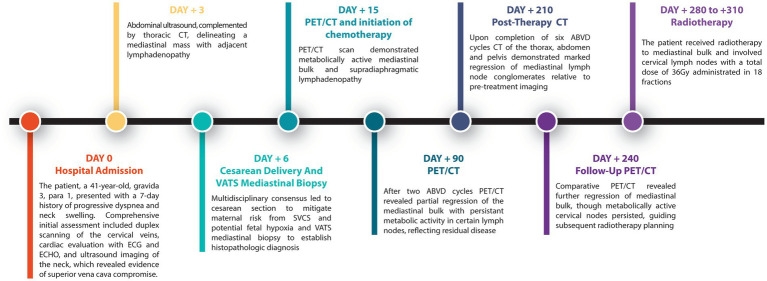
Timeline of the patient’s couse.

For an overview and better understanding of the patient’s couse, see the timeline in Figure 5.

The neonate required short-term neonatal intensive care support due to prematurity but showed normal postnatal adaptation. Follow-up demonstrated appropriate growth and neurodevelopmental milestones, with no early complications observed.

## Discussion

### Epidemiology, clinical significance and presentation of Hodgkin lymphoma in pregnancy

Hodgkin lymphoma (HL) diagnosed during pregnancy represents a rare but complex clinical entity, further complicated in this case by the development of superior vena cava syndrome (SVCS), an oncologic emergency requiring prompt recognition and intervention. The coexistence of these conditions presents a unique therapeutic challenge, necessitating a careful balance between timely maternal treatment and fetal safety within a multidisciplinary framework (6, 7).

HL is one of the most frequently encountered hematologic malignancies during pregnancy, with an estimated incidence ranging between 1 in 1,000 and 1 in 6,000 pregnancies, accounting for up to 3% of all HL cases (2, 7–9). Despite this, SVCS as an initial manifestation remains exceptionally uncommon and is typically associated with bulky mediastinal involvement, most often seen in the nodular sclerosis subtype. This rarity underscores the clinical significance of the present case and highlights the importance of maintaining a high index of suspicion when evaluating respiratory or vascular symptoms in pregnant patients.

HL diagnosed during pregnancy generally carries a prognosis comparable to that in non-pregnant patients when appropriately treated (10, 11). However, presentation with SVCS represents a medical emergency, particularly in late pregnancy, where physiological changes may exacerbate venous obstruction and compromise maternal cardiopulmonary function (3–5).

The pathogenesis of SVCS in HL is primarily related to extrinsic compression of the superior vena cava by mediastinal lymphadenopathy. This mechanical obstruction is often compounded by tumor-associated inflammatory activity, including elevated cytokine levels such as interleukin-6 (IL-6), which may contribute to systemic symptoms and disease progression (4). Importantly, physiological changes in pregnancy, including increased plasma volume and venous capacitance, may exacerbate symptom severity and obscure early clinical recognition.

Evidence from a systematic review of HL in pregnancy (12) and a multicenter Japanese cohort including both Hodgkin and non-Hodgkin subtypes (13) indicates that, while most pregnancies are not complicated by major adverse maternal or perinatal outcomes, cases with aggressive disease, such as superior vena cava syndrome, require vigilant multidisciplinary management, careful maternal-fetal monitoring, and individualized therapeutic planning.

### Management and treatment considerations of Hodgkin lymphoma during pregnancy

In non-pregnant patients, treatment of HL is standardized and stage-adapted, with ABVD-based chemotherapy representing the cornerstone of therapy, often combined with radiotherapy depending on disease stage and response.

In contrast, management during pregnancy is highly individualized and guided by three principal factors: gestational age, disease stage, and symptom burden (14, 15). According to the most recent evidence-based recommendations, including NCI PDQ (2025) and contemporary reviews, a risk-adapted approach is recommended (2, 6, 14). In early-stage, asymptomatic disease observation (“watch-and-wait”) may be appropriate, particularly when diagnosis occurs in the first trimester (15).

In symptomatic or advanced disease (including SVCS) treatment should not be delayed, as maternal risk outweighs potential fetal toxicity (14).

The therapeutic landscape of Hodgkin lymphoma has evolved significantly in recent years. While conventional regimens such as ABVD and AVD remain the backbone of treatment, targeted agents and immunotherapies, including brentuximab vedotin (anti-CD30 antibody–drug conjugate) and PD-1 inhibitors such as nivolumab and pembrolizumab have demonstrated improved outcomes in relapsed, refractory, and even frontline advanced-stage disease (2, 6, 14, 15). Notably, nivolumab combined with AVD recently received expanded frontline approval for stage III/IV classical HL, highlighting the shift toward risk-adapted and biologically targeted therapy (6, 15). Despite these advances, the use of novel agents during pregnancy remains highly restricted. ABVD continues to be considered safe after the first trimester (14), whereas brentuximab vedotin and PD-1 inhibitors lack sufficient safety data in gestation and are generally avoided unless maternal life is at imminent risk, requiring a multidisciplinary approach and close maternal-fetal monitoring (6, 14). This underscores the importance of balancing effective disease control with fetal safety in pregnant patients with high-risk or aggressive HL.

In contrast, pregnancy necessitates a more nuanced and individualized approach. Contemporary evidence supports a risk-adapted strategy, incorporating gestational age, disease burden, and symptomatology (2, 6). In carefully selected patients with early-stage, asymptomatic disease, deferral of therapy with close monitoring may be appropriate. However, in cases of symptomatic or advanced disease, particularly in the presence of SVCS, treatment should not be delayed. Vinblastine monotherapy has been utilized as a temporizing option due to its favorable safety profile, while standard ABVD chemotherapy can be administered during the second and third trimesters with acceptable maternal and fetal outcomes (6, 15).

Gestational age represents a pivotal determinant of therapeutic strategy in pregnant patients with Hodgkin lymphoma, as it directly influences both maternal management and fetal risk. Exposure to cytotoxic agents during the first trimester, particularly throughout organogenesis, is associated with a markedly increased risk of fetal malformations, miscarriage, and fetal demise; consequently, chemotherapy is generally avoided during this period. In clinically stable patients, a conservative approach with close observation may be appropriate, whereas in aggressive or life-threatening presentations, including complications such as superior vena cava syndrome (SVCS), pregnancy termination may need to be considered in order to facilitate optimal maternal treatment. In contrast, during the second and third trimesters, accumulating evidence supports the relative safety of ABVD chemotherapy, given that organogenesis is complete and the risk of major congenital anomalies is significantly reduced. Nevertheless, treatment during this stage remains associated with increased rates of preterm delivery, low birth weight, and transient neonatal myelosuppression, outcomes that are frequently related to medically indicated early delivery rather than direct fetal toxicity. Importantly, more intensive regimens, particularly those incorporating alkylating agents such as BEACOPP, are generally contraindicated throughout pregnancy due to their substantially higher teratogenic potential and overall toxicity profile.

Beyond the first trimester, accumulating evidence supports the relative safety of systemic chemotherapy, including ABVD regimens, with no significant increase in congenital malformations reported. Nonetheless, treatment remains associated with risks such as preterm delivery and low birth weight, often driven by medically indicated early delivery rather than direct drug toxicity (16, 17).

The prognosis of Hodgkin lymphoma diagnosed during pregnancy is remarkably favorable and, when managed appropriately, closely parallels that observed in age-matched non-pregnant patients, with overall survival rates consistently exceeding 90% (15, 16).

From a fetal perspective, outcomes are generally reassuring, particularly when systemic therapy is initiated beyond the first trimester, once organogenesis has been completed and the risk of major congenital anomalies is substantially reduced. The most frequently reported complications include prematurity and low birth weight; however, these are often attributable to iatrogenic early delivery driven by maternal indications rather than direct cytotoxic effects of chemotherapy (16, 17). Importantly, long-term follow-up studies of children exposed to chemotherapy in utero have not demonstrated significant impairments in physical development, neurocognitive function, or increased oncologic risk, although the available data remain limited and underscore the need for continued longitudinal surveillance (17).

Collectively, these findings support the concept that, with careful multidisciplinary management and judicious treatment selection, excellent maternal and fetal outcomes can be achieved, even in the context of a complex and potentially life-threatening diagnosis such as HL during pregnancy.

Pregnancy complicated by HL represents one of the most formidable challenges in onco-hematology, as clinicians must simultaneously safeguard maternal life and optimize fetal outcomes. In standard practice for non-pregnant patients, first-line therapy with ABVD (adriamycin, bleomycin, vinblastine, dacarbazine) is highly efficacious, achieving excellent overall and progression-free survival. However, during gestation, particularly in the first trimester, ABVD is associated with potential teratogenicity and long-term fetal risks, although administration in the second and third trimesters appears reasonably safe. In this context, vinblastine monotherapy has emerged as a valuable alternative: its high protein-binding properties and interaction with P-glycoprotein substantially limit placental transfer, allowing disease control in clinically stable patients without imposing significant fetal risk (6, 15).

Reports of HL presenting with SVCS during pregnancy are scarce, with most cases involving bulky mediastinal disease diagnosed in the second or third trimester. Available literature supports the feasibility of individualized management strategies incorporating observation, vinblastine monotherapy, or ABVD chemotherapy, depending on disease severity and gestational timing (2, 6).

Notably, recent data suggest that conservative approaches, when clinically appropriate, may achieve excellent maternal and fetal outcomes, with reported survival rates approaching 100% in selected cohorts (6, 15). The present case contributes to this limited body of evidence by emphasizing the importance of early recognition and timely, tailored intervention in managing this rare but potentially life-threatening presentation.

Nonetheless, certain clinical scenarios preclude expectant management or monotherapy. Superior vena cava syndrome (SVCS) due to a bulky mediastinal mass represents a true oncologic emergency, with immediate threats to both maternal and fetal wellbeing. Compression of the thoracic vasculature may precipitate maternal dyspnea, orthopnea, and diaphragmatic compromise, while fetal hypoxia can ensue secondary to maternal hemodynamic instability. In these circumstances, a judiciously timed cesarean section may become the safest therapeutic pivot. Several reports illustrate this approach: Hagège et al. describe a third-trimester patient with SVCS who underwent urgent cesarean delivery, subsequently allowing the initiation of ABVD with no compromise to maternal or neonatal outcomes; Lishner et al. (1992) second-trimester cases indicate that delivery may be prioritized, followed by vinblastine monotherapy and subsequent ABVD, while early clinical observations further support the principle that maternal stabilization through delivery enables timely and safe definitive therapy without compromising survival.

Other modalities, including corticosteroids and radiotherapy, play a limited role during pregnancy. Corticosteroids may offer short-term symptom relief but are not curative, and radiotherapy carries significant teratogenic potential and is typically deferred until postpartum (18).

In the present case, urgent cesarean delivery was warranted due to progressive SVCS, maternal respiratory compromise, and imminent risk of fetal hypoxia. This intervention allowed safe postpartum initiation of ABVD therapy in accordance with current international guidelines, striking an optimal balance between maternal disease control and fetal preservation. This strategy exemplifies a key principle in onco-obstetrics: when maternal life or fetal viability is acutely threatened, prompt delivery can be a life-saving bridge to definitive therapy, yielding favorable outcomes for both mother and child.

Disease stage remains the most critical determinant of management strategy. Early-stage disease (IA–IIA) may allow for deferred treatment or less intensive approaches, whereas advanced-stage disease (IIB–IV) typically necessitates immediate systemic therapy regardless of pregnancy status (6, 16, 17). Histological subtype also plays a relevant role, with nodular sclerosis HL being the most commonly associated with mediastinal masses and thus SVCS, as observed in the present case.

In the present case, urgent delivery was favored over antepartum chemotherapy due to severe SVCS-related symptoms, maternal risk, and advanced gestational age. Performing cesarean delivery and VATS biopsy during a single operative session minimized diagnostic delay and enabled rapid initiation of definitive oncologic therapy.

### Staging and imaging considerations during pregnancy

Standard staging in HL relies on PET/CT imaging; however, its use is generally contraindicated during pregnancy due to fetal radiation exposure. Current guidelines recommend MRI and ultrasound as safer alternatives, although these modalities may have reduced sensitivity for detecting small-volume disease (19–22). Consequently, staging in pregnant patients may be less precise, and clinical judgment assumes a more prominent role in management decisions.

Surveillance strategies also differ significantly. In pregnant patients, follow-up relies primarily on clinical examination and non-ionizing imaging modalities, whereas non-pregnant patients benefit from standardized PET/CT-based response assessment.

In the present case, computed tomography (CT) of the thorax was performed despite pregnancy, in preference to magnetic resonance imaging (MRI), due to the urgent need to exclude pulmonary embolism (PE), a life-threatening condition that can mimic or coexist with superior vena cava syndrome (SVCS). While MRI is generally preferred in pregnancy to minimize fetal radiation exposure, CT pulmonary angiography remains the gold standard for PE diagnosis due to its high sensitivity and specificity (19, 20).

Guidelines from the American College of Radiology (ACR) and the Society of Thoracic Radiology indicate that CT of the chest can be safely performed during pregnancy when clinically indicated, particularly when the benefits of prompt and accurate diagnosis outweigh the theoretical risks of fetal radiation. The estimated fetal dose from a standard chest CT is low (<0.1 Gy) and is well below thresholds associated with teratogenicity or increased cancer risk (20, 21). Protective measures, including abdominal shielding and dose modulation, were employed in this case to minimize exposure.

MRI without contrast was considered; however, MRI has lower sensitivity for detecting pulmonary emboli compared to CT pulmonary angiography, especially in the acute setting. Therefore, in symptomatic pregnant patients with suspected PE, CT remains the imaging modality of choice, consistent with contemporary guidelines (20, 21).

This approach underscores the importance of individualized, risk–benefit–driven imaging decisions in pregnant patients presenting with acute thoracic symptoms, ensuring maternal safety while minimizing fetal exposure.

### Immunologic considerations and cytokine-mediated disease progression

Pregnancy is associated with dynamic immunologic adaptations, including increased levels of pro-inflammatory cytokines such as interleukin-6 (IL-6), which play critical roles in placental development and fetomaternal immune tolerance (23–25). In Hodgkin lymphoma, IL-6 promotes survival and proliferation of Reed–Sternberg cells through activation of the JAK/STAT3 signaling pathway and has been correlated with disease burden, systemic symptoms, and prognosis (23, 24, 26–28).

Although IL-6 levels were not measured in this patient, the unusually rapid tumor progression observed during the third trimester raises the hypothesis that pregnancy-associated cytokine shifts may have contributed to disease acceleration. While speculative, this hypothesis is biologically plausible and consistent with existing literature. Importantly, it does not alter current management but underscores the need for heightened clinical vigilance and further investigation into the immunologic interplay between pregnancy and lymphoproliferative malignancies.

### Therapeutic considerations and clinical challenges in lymphoma during pregnancy

HL is more commonly encountered during pregnancy and typically presents as a mediastinal mass with relatively indolent progression, allowing planned chemotherapy (ABVD) after the first trimester with favorable maternal and fetal outcomes (6, 16). In contrast, non-Hodgkin lymphoma (NHL) is rarer but often more aggressive, with rapid tumor growth and higher risk of acute complications such as superior vena cava syndrome. Management of NHL requires urgent, multidisciplinary intervention, often using CHOP-based regimens in the second and third trimesters, with maternal prognosis dependent on subtype and timing of therapy (16, 29).

While HL allows more predictable treatment scheduling and monitoring, NHL may necessitate immediate therapy to prevent life-threatening maternal compromise. Both conditions highlight the importance of balancing maternal stabilization, fetal safety, and timing of delivery, emphasizing the critical role of multidisciplinary care in optimizing outcomes.

Although our patient was diagnosed with classical Hodgkin lymphoma, her clinical course was unusually aggressive, resembling the rapid progression more typical of non-Hodgkin lymphoma. She presented with severe superior vena cava syndrome requiring urgent intervention, highlighting that histologic subtype alone may not fully predict disease behavior during pregnancy. Elevated cytokine activity, particularly interleukin-6 (IL-6), likely contributed to the systemic inflammatory response, edema, and exacerbation of venous obstruction (16, 30). This underscores the importance of early recognition and prompt multidisciplinary management, even in cases of HL that histologically appear indolent.

While HL generally allows for planned chemotherapy with predictable outcomes, aggressive presentations, like in our case, blur the clinical distinctions between HL and NHL, necessitating urgent intervention, careful maternal-fetal monitoring, and flexible treatment planning (29, 30).

### Clinical considerations and safety of breastfeeding during chemotherapy for Hodgkin lymphoma

Breastfeeding in the context of Hodgkin lymphoma (HL) treatment represents a critical yet often under-discussed aspect of postnatal management. Current evidence and international guideline recommendations strongly advise against breastfeeding during active chemotherapy due to the excretion of cytotoxic agents into breast milk and the potential for significant neonatal toxicity (16, 31).

The standard ABVD regimen, commonly employed in the treatment of HL, includes doxorubicin, bleomycin, vinblastine, and dacarbazine-agents with documented or theoretical transfer into breast milk. Doxorubicin, an anthracycline, is of particular concern due to its potential for cumulative cardiotoxicity and myelosuppression, while vinblastine, a vinca alkaloid, may interfere with microtubule formation and cellular division, posing a risk of profound bone marrow suppression and neurotoxicity in the neonate. Dacarbazine, an alkylating agent, carries mutagenic and carcinogenic potential, raising additional concerns regarding long-term safety. Although data on bleomycin excretion are limited, its known pulmonary toxicity further supports a precautionary approach (31, 32).

Reported and theoretical adverse effects in breastfed infants exposed to chemotherapeutic agents include neutropenia, immunosuppression, impaired growth, gastrointestinal toxicity, and potential long-term oncogenic risk. Given these concerns, breastfeeding is considered contraindicated throughout the duration of systemic chemotherapy (16, 31, 32).

Following completion of therapy, breastfeeding may be considered; however, this should only occur after an adequate drug clearance (“washout”) period, which varies depending on the pharmacokinetics of individual agents. At present, there is no universally established safe interval, and decisions should be individualized, incorporating oncologic, pharmacologic, and pediatric expertise (31).

Importantly, early counseling regarding breastfeeding should be an integral component of management, allowing patients to make informed decisions and consider alternatives such as milk expression and disposal during treatment. This approach ensures neonatal safety while supporting maternal autonomy and optimizing overall care in this complex clinical setting.

## Conclusion

This case underscores the critical importance of early recognition and multidisciplinary management of superior vena cava syndrome caused by Hodgkin lymphoma during pregnancy. Urgent delivery combined with immediate diagnostic intervention enabled timely initiation of chemotherapy and resulted in favorable maternal and neonatal outcomes. The potential role of pregnancy-related cytokines such as IL-6 in accelerating lymphoma progression remains hypothetical but merits further investigation.

## Data Availability

The raw data supporting the conclusions of this article will be made available by the authors, without undue reservation.
